# Preventing Preterm Birth: The Search for Tocolytic Synergism

**DOI:** 10.1007/s43032-025-01941-4

**Published:** 2025-08-25

**Authors:** Md Reduanul Hossain, Marina Paul, Jorge M. Tolosa, Roger Smith, Jonathan W. Paul

**Affiliations:** 1 College of Health, Medicine and Wellbeing, University of Newcastle, Callaghan, NSW 2308 Australia; 2 Mothers and Babies Research Program, Hunter Medical Research Institute, 1 Kookaburra Circuit, New Lambton Heights, NSW 2305 Australia; 3 John Hunter Hospital, New Lambton Heights, NSW 2305 Australia

**Keywords:** Preterm labor, Tocolytic, Drug combination, Synergism, Additivity, Antagonism, Quantitation, Pharmacological model

## Abstract

Preterm birth is a common cause of neonatal mortality and morbidity, which are increasingly prevalent today. Preterm birth affects millions of babies worldwide and is the subject of intense study in its pathophysiology and therapy. Tocolytic agents are drugs used to prevent preterm birth and prolong pregnancy, to allow the fetus to remain *in utero* for further maturation, permit antenatal corticosteroid treatment to accelerate fetal lung maturity, and allow enough time for *in uter*o-transfer of the baby to a tertiary care facility. However, the application of traditional tocolytic therapy is often limited due to maternal and fetal side effects. As such, there is a pressing need for safer and more effective tocolytic therapy. Combinational tocolysis has the potential to improve tocolytic efficacy and reduce side effects. A limited number of papers have revealed the prospective benefits of combinational tocolysis by drug synergism and reduced drug toxicity. This review summarizes the current landscape of combinational tocolysis directed at achieving tocolytic synergism and examines the quantitative methods utilized for detecting synergistic tocolytic combinations.

## Introduction

Preterm birth (PTB) is defined as birth before 37 completed weeks of gestation and is a significant determinant of neonatal mortality and morbidity [1, 2]. It is estimated that 15 million babies are born prematurely every year [1], and approximately 1 million children fail to survive PTB-related complications each year before they reach their fifth birthday [3]. Across the world, the rate of PTB ranges from 5 to 18% and is increasing in many countries [1]. PTB leads to serious health complications because the fetus’ bodily organs, such as the lungs and brain, are under-developed at the time of birth, as significant organ development occurs in the final weeks before term parturition. As a result, surviving newborns start their new life at significantly greater risk of physical and psychological difficulties, which translates into both short- and long-term morbidities [1]. Among the several obstetric precursors, spontaneous preterm labor (sPTL) is the leading cause of PTB, accounting for approximately 45% of cases, with idiopathic sPTL comprising roughly half of these cases. Certain conditions increase the risk of PTL, including infection or inflammation (a major cause of preterm premature rupture of the membranes (PPROM), which is also responsible for approximately 25% of PTB [1]), uteroplacental ischemia or hemorrhage, uterine overdistension, as well as maternal stress and other immunologic-mediated processes [4]. Each can lead to dysregulation of uterine contractility and may ultimately result in PTB.

Drugs used to relax the uterus are called tocolytics and may be deployed in cases of threatened sPTL to prevent or delay PTB. The primary purpose of tocolytic therapy is to prolong pregnancy as close to term as possible, ideally allowing the fetus to remain in utero for further maturation. In current practice, tocolytics often achieve a delay of up to 48 h, which provides time for antenatal corticosteroid treatment to improve fetal lung maturation and for maternal transfer to a care facility equipped with a specialist neonatal intensive care unit (NICU) [5].

Tocolytics work by either blocking contraction-promoting signaling pathways or activating relaxation-promoting signaling pathways within uterine myocytes. Several classes of therapeutics have been included in tocolytic regimens for their ability to block contractions or promote uterine relaxation. These include beta-mimetics (e.g., salbutamol, ritodrine, and fenoterol), calcium ion (Ca^2+^) channel blockers (CCBs) (e.g., nifedipine and nicardipine), Ca^2+^ competitors (e.g., magnesium sulfate/gluconate), non-steroidal anti-inflammatory drugs (NSAID) (e.g., indomethacin, celecoxib, and rofecoxib), oxytocin (OT) receptor antagonists (OTRA) (e.g., Atosiban and Nolasiban), nitric oxide donors (e.g., nitroglycerine), and phosphodiesterase (PDE) inhibitors (e.g., rolipram and aminophylline). There are also novel drugs that have recently been assessed for their relaxant effects on ex vivo human myometrium. These include the non-specific inhibitor of inositol triphosphate receptors (IP_3_R), 2-aminoethoxydiphenyl borate (2-APB) and the Rho-A kinase (ROCK) inhibitor, Glycyl-H-1152 (GH) [6, 7]; S-nitrosoglutathione reductase (GSNOR) inhibitors, N6022 [8]; connexin 43 (Cx43) inhibitors, 18β-glycyrrhetinic acid and nebivolol [9]; Beta 3-adrenergic receptor (β_3_-AR) agonists mirabegron [10]; hydrogen sulphide (H_2_S)-releasing compound, ATB-346 [11]. These tocolytic agents possess different modes of action in that some block the actions of uterotonins, such as indomethacin, mirabegron and Atosiban, whereas others prevent the availability and actions of intracellular Ca^2+^ within myocytes, such as nifedipine, salbutamol, and magnesium sulphate. The use of individual tocolytics and their effectiveness in preventing PTB have already been discussed in multiple reviews [12–14].

While short-term tocolysis may have a role in improving PTB outcomes, there is no single agent currently available as a first-line tocolytic that is safe for the mother and fetus [15]. Moreover, all currently available tocolytics have relatively limited efficacy, with the ability to delay delivery for only short periods, ranging from a few days to a week. The contributing factors are, but not limited to, the lack of specificity of tocolytics, with effects on organs and tissues other than the myometrium that result in unfavorable feto-maternal side effects, and the possible interpatient variability to drug response, which means certain drugs may not be equally effective across different population groups of varying gestational ages. Another contributing factor is the multifactorial mechanisms of PTL itself, which are not all addressed via the administration of a single tocolytic (monotherapy). In recognition of these shortfalls, there has been a spate of new approaches seeking therapeutic intervention with improved efficacy and reduced toxicity for PTL management. An emerging approach, and the focus of this review, is the concept of *combinational tocolysis*, which involves administering a combination of two or more tocolytics for blocking myometrial contractions.

### Combinational Tocolysis

To date, both preclinical and clinical evaluations of tocolytic therapy for PTL have primarily focused on single agents that target an individual contraction signaling pathway within uterine myocytes. However, sPTL is a complex pathophysiology underpinned by multifactorial contributing mechanisms [16]. Therefore, tocolytic monotherapy (administration of a single agent) may not overcome all the contributing mechanisms/factors driving PTL in a pregnant woman. Indeed, achieving clinically effective tocolysis is probably dependent on successfully manipulating several signaling pathways simultaneously. Moreover, the outcomes from preclinical and clinical studies reporting the limited efficacy of different tocolytics when deployed as monotherapies suggest that the PTL should be confronted on several fronts [17–23].

**Fig. 1:**
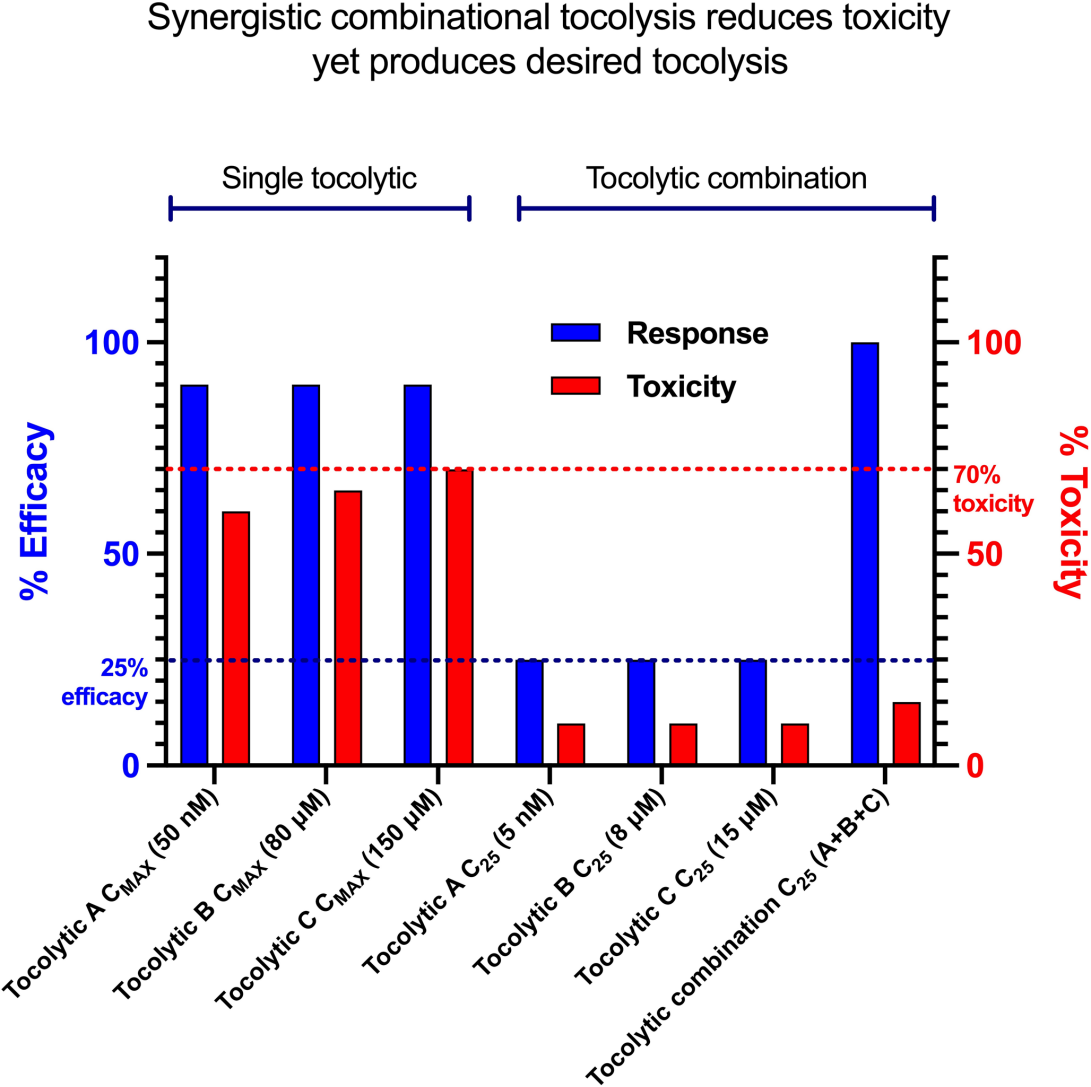
Schematic representation shows the benefits of combinational tocolysis: Tocolytics A, B, and C show 90% efficacy (as an example) when used alone at their respective C_MAX_ and trigger severe toxicity (e.g., 70%). But as a synergistic combination, the same efficacy, or even greater (100%), can be achieved at reduced tocolytic concentrations (e.g., C_25_), which reduces toxicity (e.g., 15% or less). Reproduced from Sun et al. [112] *C*_*MAX*_, a concentration that produces maximum possible efficacy; *C*_*25*_, a concentration that produces 25% efficacy

Combinational tocolysis can improve the tocolytic safety profile, and where the simultaneous targeting of multiple pro-contraction pathways is included, can also improve tocolytic efficacy [17]. This is because the simultaneous blocking of multiple contraction pathways (by combining tocolytics) can allow therapeutic efficacy (contraction inhibition) to be achieved at reduced drug concentrations and with reduced off-target side effects compared to monotherapy, thus improving patient safety. Both synergistic and additive effects contribute to these benefits, with synergy offering greater-than-expected efficacy and additivity providing reliable inhibition at lower doses, reducing toxicity. In that way, maternal safety is improved as the amount of drug that reaches non-target organs is reduced, reducing the prevalence and severity of maternal side effects (Fig. 1). Combinational tocolysis can also improve fetal safety by reducing drug administration, which reduces transplacental passage of drugs, thereby reducing the risk of fetal toxicity. Moreover, combination treatment may have the added benefit of overcoming interpatient drug response variability, as there is a greater chance of at least one of the drugs producing the desired clinical effect [24]. Given these therapeutic benefits, combinational therapeutic approaches have been applied to a wide range of disease conditions, including cancer [25–27] and infectious diseases, such as AIDS [28–30], tuberculosis [31, 32], and malaria [33, 34]. However, treating premature uterine contractions by combinational tocolysis during pregnancy is in its infancy.

### Quantification of Drug Combinations and Reference Models to Detect Synergy

**Fig. 2:**
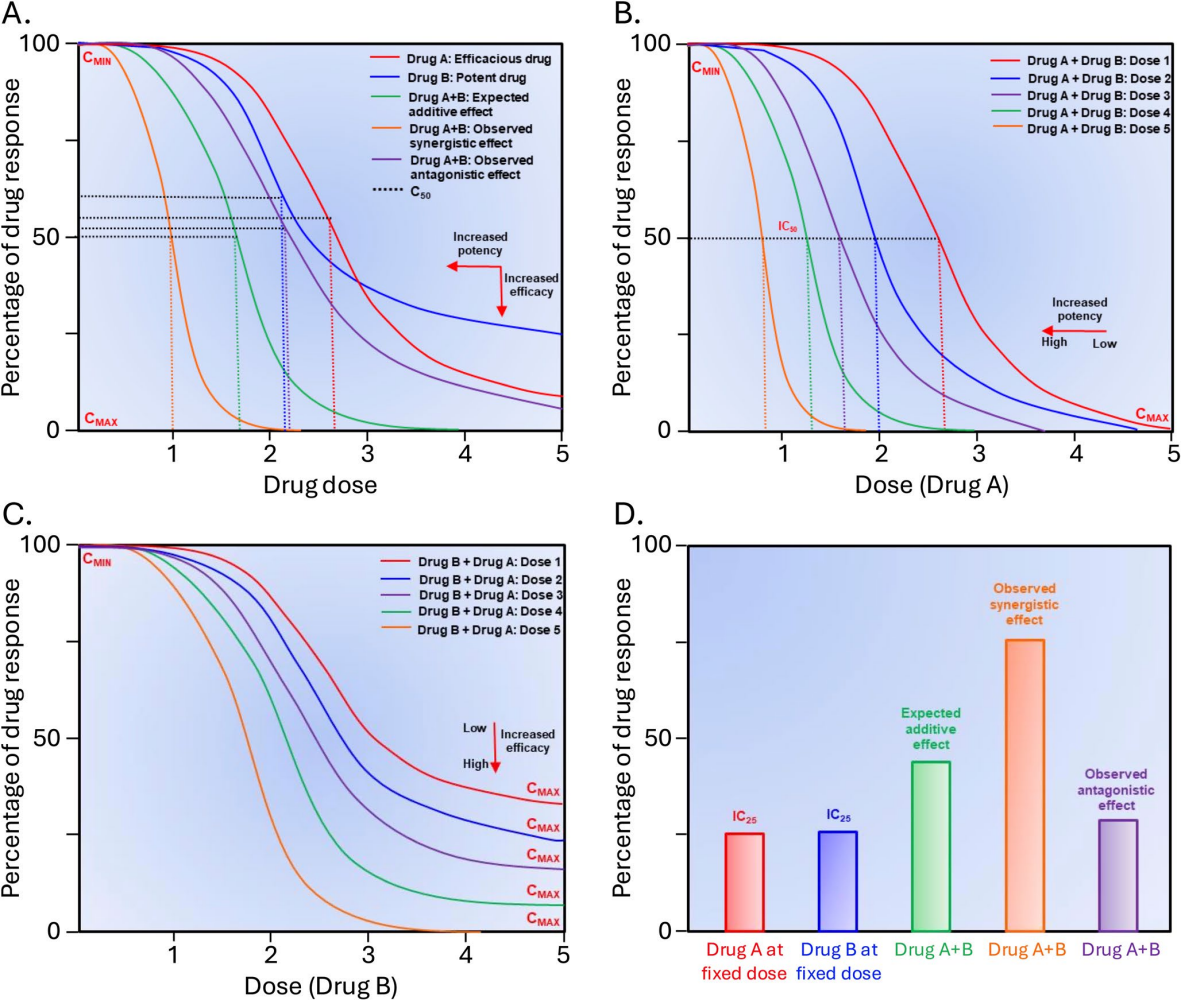
Schematic illustration of quantitative analysis of drug combination results. (**A**) the representative dose-response curves and associated C_max_ and C_50_ for example Drug A and Drug B. A left shift of curve indicates increased potency whereas a down shift of curve indicates increased efficacy. Hence, Drug A is more efficacious (higher C_max_) than Drug B (lower C_max_), but Drug A is less potent (higher C_50_) than Drug B (lower C_50_). An expected additive effect is calculated by applying a reference model of choice for quantitative analysis of drug combination, A + B based on their individual responses across different drug doses. Alteration of the dose-response curve (i.e., lower/higher C_50_/C_max_) for an expected additive effect indicates synergism or antagonism depending on the degree/pattern of shift, i.e., if the experimentally observed effect curve shifts left or right (potency changes) or/and down or up (efficacy changes) in compared to the expected additive effect curve then the combined effect is termed as synergistic and antagonistic, respectively. Hence based on the pattern of curve shifting, the synergism and antagonism can be potency (**B**) or efficacy dependent (**C**), or both. Minimum/statistically insignificant change from the expected additive effect curve indicates additivity. The results can be further confirmed by a bar graph with selected concentrations (**D**). *C*_*MIN*_ and *C*_*MAX*_, a concentration that produces minimum and maximum possible efficacy, respectively; *C*_*50*_, a concentration that produces 50% efficacy

The therapeutic effects produced by multiple drugs delivered in combination can be defined as additive, synergistic, or antagonistic. An additive effect is a theoretically expected effect whereby the response resulting from a combination is equal to the sum of the individual responses of constituent drugs if they had acted independently; this is generally considered as the hypothetical null effect for synergy or antagonism detection methods. To quantify the interaction between drugs, the observed combination response of multiple drugs is compared to the expected/additive (null) effect under the assumption of non-interaction predicted by a reference model. Any significant deviation from the expected/additive effect would be categorized as either synergism when the effect of the combination is significantly greater than the expected (additive) effect, or antagonism when the effect of the combination is significantly less than the expected (additive) effect [35–37]. It is important to note that different nomenclatures have been developed, and synergism has also been termed ‘*potentiation*’, ‘*augmentation*’, ‘*super additivity*’, and ‘*supra-additivity*’ [37, 38]. Moreover, synergy can be efficacy-driven, to achieve an effect that is significantly greater than the additive effect, or potency-driven, to achieve a clinically desired effect (target efficacy) but with reduced doses of constituent drugs (dose reduction), or both (Fig. 2) [38, 39].

**Fig. 3:**
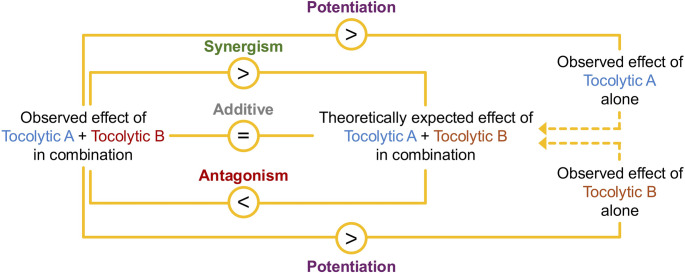
Overview of statistical comparisons for confirming drug combination outcomes of synergism, additivity, antagonism, or potentiation. The sign inside the circle indicates the outcomes of the comparisons of contraction-blocking potency (*>*, greater than; =, equal to; *<*, less than)

For clarity, this review has utilized the term ‘synergism’ to refer to situations where quantitative analyses to detect synergism have been performed, and the observed inhibition was determined to be significantly greater than the expected inhibition. Where quantitative analyses to detect synergism have not been performed, the term “potentiation” is used and indicates situations where statistical analyses (i.e. ANOVA) have confirmed that a tocolytic combination was more effective at inhibiting contractility than the constituent tocolytics alone, but not relative to an expected additive effect (see Fig. 3).

There are numerous reference models proposed for detecting additive drug interactions, and essentially, three popular reference models are extensively reported in the literature [37, 38]: the Highest Single Agent (HSA) model [40], the Loewe Additivity model [41], and the Bliss Independence model [42]. The HSA and Bliss Independence models follow an effect-based strategy that compares the effect of a combination (observed response) directly to the effects of its constituent drugs (expected additive effect), which constitutes efficacy-driven synergy [38, 39, 43]. In contrast, the Loewe Additivity model uses a dose-effect-based strategy, where the expected additive effect of a drug combination depends on the individual drugs’ dose-response curve to find the required amount or concentration of each drug that produces the same quantitative effect, which constitutes potency-driven synergy [38, 39].

Based on these reference models, and many of their subsequent variants and extensions, several quantitative methods have been developed and extensively used to measure the effects of drug combinations [44–46]. These include the Combination Index [47], Fractional Effect Analysis [48], Isobologram Analysis [41], Interaction Index [49, 50], Median Drug Effect Analysis [51], Multi-dimensional Synergy of Combinations (MuSyC) [52], Zero Interaction Potency (ZIP) [53], Effective Dose Model [54], Partial Differential Equation (PDE) Hill model by Schindler [55], Bivariate Response to Additive Interacting Doses (BRAID) model [56], and more. Several online software tools have emerged for analyzing high-throughput combinational data, including SynergyFinder Plus [57], MuSyC [52], SynToxProfiler [58], Combenefit [59], CompuSyn, and SiCoDEA [60]. However, considerable debate remains regarding the method of choice and there is still no universally accepted guideline for choosing the optimal reference model [35, 61, 62].

Most of the preclinical studies reviewed lack quantitative assessment to confirm a synergistic or additive effect. As such, for the present review, where possible the authors have extracted relevant data from the reviewed studies and conducted retrospective quantitative analyses via the Bliss Independence model.

### A Review of Combinational Tocolysis

The ensuing review of tocolytic combinations is divided into the examination of preclinical (in vitro, ex vivo, in vivo) and clinical studies.

### Beta-mimetics in Combination with Calcium Channel Blockers

The combination of beta-mimetics and CCBs is one of the more extensively tested combinations for tocolytic therapy, and synergistic effects from such combinations have been observed in different experimental settings.

#### Ex Vivo Animal Studies

Gallagher et al. [63]. evaluated the effect of nifedipine and ritodrine in combination against contraction inhibition in pregnant rat myometrial strips. The study showed that administering a single dose of ritodrine (0.144 µg) + nifedipine (0.5 µg) in combination reduced both the area under the curve (AUC) (89.8%) and the frequency of OT-stimulated contractions to a significantly greater degree than ritodrine or nifedipine alone (AUC reduction; 26% for ritodrine and 78.4% for nifedipine) [63]. The authors did not assess whether this was an additive or synergistic effect, however, our retrospective application of the reported data using the Bliss Independence model indicated that ritodrine + nifedipine in combination was synergistic, in that the observed inhibition (89.8%) was significantly greater than the theoretically expected inhibition at the same concentration (83%), which was calculated from the sum of the individual drug effects [63]. Hajagos-Tóth et al. [64]. evaluated the effect of terbutaline + nifedipine in combination against potassium chloride (KCl)-induced contractions in rat pregnant myometrial strips. The study showed that terbutaline + nifedipine in combination reduced contraction AUC to a greater extent than the single agents alone, indicating potentiation. Interestingly, the study reported that the magnitude of potentiation was dependent on the order of nifedipine and terbutaline administration, in that the degree of potentiation observed, when a single dose of nifedipine (10^− 7^ M) was administered before cumulative administration of terbutaline (10^− 10^– 10^− 4^ M), was greater than the degree of potentiation observed, when a single dose of terbutaline (10^− 7^ M) was administered before the cumulative administration of nifedipine (10^− 11^– 10^− 6^ M) [64]. In contrast, Doret et al. [20] found that against contracting pregnant rat myometrial strips, ritodrine (3.1 × 0^− 7^ M) + nicardipine (5.8 × 10^− 9^ M) in combination produced an additive effect, as determined by the Bliss Independence model, in that there was no significant difference between expected AUC inhibition (75%, which was calculated from the sum of the individual drug effects) and observed AUC inhibition (∼77%) [20]. However, the observed combined effect (∼77%) was more potent than the effect of each constituent drug alone (50%) at the same concentration [20].

#### Ex Vivo Human Studies

Saade et al. [65] showed that ritodrine (4.4 × 10^− 4^ M) + nifedipine (4.2 × 10^− 5^ M) in combination inhibited KCl-induced contractions from laboring and non-laboring human myometrial strips to a significantly greater degree than either of the single tocolytics alone at the same concentration, indicating a potentiation effect. Similarly, Carvajal et al. [66]. demonstrated a potentiation effect of ritodrine + nifedipine in combination against OT-induced contractions in pregnant human myometrial strips. The study examined the effects of cumulative concentrations of ritodrine (5 × 10^− 8^– 5 × 10^− 5^ M) and nifedipine (1 × 10^− 10^– 5 × 10^− 6^ M) alone and in combination and reported that the contraction-inhibitory effect of the combination was significantly greater than the single agents alone at mid-range concentrations, and shifted the concentration-response curve to the left, indicating a potentiation effect. Moreover, Hajagos-Tóth et al. [67] showed that terbutaline (10^− 7^ M) + nifedipine (10^− 11^– 10^− 5^ M) or nifedipine (10^− 7^ M) + terbutaline (10^− 11^– 10^− 5^ M) in combination inhibited OT (10^− 6^ M)-induced contractions in pregnant human myometrial strips and potentiated each other’s myometrial relaxation effects, which were significantly greater compared to the single agents alone. The result was consistent with the prior study in rat myometrial strips by Hajagos-Tóth et al. [64], and the magnitude of potentiation was also dependent upon the sequential administration of nifedipine first, followed by terbutaline.

#### In Vivo Animal Studies

Hajagos-Tóth et al. [67] administered nifedipine (3.89 mg/d) + salmeterol (0.13 mg/d) in combination, on fetal gestation days 16 and 18, and delayed RU486-induced PTB in rats. This was a significantly greater extent than nifedipine treatment alone at the same concentration (24 versus (vs.) 6.6 h, respectively). The effect of salmeterol alone was not assessed, nevertheless, the authors concluded that nifedipine + salmeterol exhibited a potentiation effect in vivo. However, the potentiation effect was not confirmed via the application of an assessment model.

#### Human Clinical Studies

In a retrospective study of 213 pregnancies with PTL (gestational age (GA) range, 20–34 weeks), Kim et al. [68] compared the use of ritodrine + nifedipine in combination (104 women) vs. ritodrine alone (109 women) for maternal side effects and PTL suppression. The study reported that ritodrine + nifedipine in combination was more effective at delaying birth for over seven days than ritodrine alone at the same dose used for the combination treatment (pregnancy prolongation for 7 days, 72.1% vs. 59.6%, respectively), and both treatments were associated with mild maternal side effects [68]. However, a prospective cohort study recommended the sole use of nifedipine, as opposed to use in combination with salbutamol, for PTL management [69]. The prospective cohort study was carried out on 76 women (GA range, 24–35 weeks) with threatened PTL who were divided into two treatment groups (38 women in each group); those who received nifedipine alone (10 mg loading dose of oral nifedipine every 15 min for 1 h and a maintenance dose of 20 mg four times a day) (mean gestational age (MGA), 30.9 weeks) and those who received salbutamol (0.01 mg/ml intravenous (IV) infusion) + nifedipine (same regimen as the single treatment) in combination (MGA, 31 weeks). The authors reported a statistically significant difference (*p* < 0.05) between the delay in labor achieved by nifedipine alone (6.74 weeks, on average) compared to salbutamol + nifedipine in combination (5.21 weeks, on average). Nifedipine alone was also associated with fewer maternal side effects (*p* < 0.001). As such, the authors recommended nifedipine monotherapy over salbutamol + nifedipine in combination for the purpose of delaying PTB [69].

### Beta-mimetics in Combination with Non-steroidal Anti-inflammatory Drugs

#### Ex Vivo Animal Studies

Doret et al. [19] examined the effects of ritodrine + rofecoxib (a selective prostaglandin-endoperoxide synthase 2 (PTGS2) inhibitor) in combination against contracting pregnant rat myometrial strips ex vivo. The study reported that ritodrine (3.1 × 10^− 7^ M) + rofecoxib (4.8 × 10^− 9^ M) in combination was synergistic, in that the observed inhibition of contraction AUC (93%) was significantly greater than the effect of either agent alone (50%) at the same concentration and the expected additive inhibition (75%), as determined by Bliss Independence model.

#### Human Clinical Studies

A prospective and double-blind study compared the effectiveness of ritodrine alone (10 mg four times per day orally/intramuscularly (IM) until GA 38 weeks or labor occurred) vs. ritodrine (at same dosing regimen as single treatment) + indomethacin (50 mg suppository three times per day rectally for 2 weeks or until labor occurred) in combination in a clinical setting. The study was conducted on two randomized control groups, where 22 pregnant women with threatened PTL were included in each group (GA range, 20–36 weeks, MGA for combination and ritodrine groups were 30.6 and 30.5 weeks, respectively). The study found that ritodrine + indomethacin in combination was significantly more effective than ritodrine alone at prolonging pregnancy (41.3 days vs. 25.9 days, *p* < 0.005), without any unfavorable side effects on the fetus [70].

### Beta-mimetics in Combination with Magnesium

#### Ex Vivo Human Studies

In an ex vivo study of contracting pregnant human myometrial strips, Saade et al. [65] compared the effectiveness of magnesium sulfate (2.1 × 10^− 2^ M) in combination with either ritodrine (4.4 × 10^− 4^ M) or terbutaline (7 × 10^− 4^ M). Both combinations, magnesium sulfate + ritodrine and magnesium sulfate + terbutaline, were found to be more effective than the single agents alone (at the same concentrations) at inhibiting ex vivo myometrial contractions (AUC), thus indicating that both combinations resulted in potentiation.

#### Human Clinical Studies

Several clinical studies have been conducted to evaluate the effectiveness of beta-mimetics in combination with magnesium sulfate in humans. In a randomized control trial (RCT) of 128 pregnant women with threatened PTL (GA range, 28–35 weeks, MGA for combination and magnesium sulfate group was 29.4 and 28.6 weeks, respectively), Shuifang et al. [71] reported that ritodrine (infused at 2 g/h for 12 h) + magnesium sulfate (infused at 0.005 mg/h up to 0.35 mg/h for 12 h) in combination reduced the incidence of severe side effects and improved pregnancy outcomes and neonatal neurological functions, compared to magnesium sulfate alone. However, the pregnancy prolongation achieved by the combination treatment was significantly shorter than the prolongation achieved by magnesium sulfate alone at the same dose (21.5 vs. 28.3 h on average, respectively) (*p* < 0.05) [71]. Another RCT by Ferguson et al. [72] also reported that ritodrine + magnesium sulfate in combination did not improve tocolytic efficacy compared to ritodrine alone at the same dose (pregnancy prolongation by 11.5 vs. 16.8 days on average, respectively) and resulted in more severe cardiovascular side effects [72]. The study was conducted on 50 pregnant women with threatened PTL and randomized 28 women into the ritodrine + magnesium sulfate combination group (MGA, 30.6) and 22 women into the ritodrine alone group (MGA 29.6). However, Diamond et al. [73] argued that the cardiovascular effects reported by Ferguson et al. [72] were likely attributable to the infusion rate of magnesium sulfate.

### Beta-mimetics in Combination with Progesterone

#### In Vivo Animal Studies

Gálik et al. [74]. administered salmeterol (130 µg/d per animal), progesterone (0.5 mg per 0.1 mL/d), or salmeterol + progesterone in combination (at the same concentrations as the single drug treatments) to pregnant rats on fetal gestation days of 15–18 and found that the combination treatment delayed PTL by 5.2 h on average, compared to a 2.4 h delay by salmeterol alone and a 0.5 h delay by progesterone alone. These results indicate potentiation. However, the potentiation effect was not confirmed via the application of an assessment model.

#### Human Clinical Studies

A prospective, double-blind study by Noblot et al. [75] examined the tocolytic efficacy of progesterone on 44 pregnant women (GA, 30–33 weeks) with threatened PTL as a supplement to ritodrine treatment. The study showed no significant differences between ritodrine (total dose 345 mg) + progesterone (400 mg every 6 h during the first 24 h, 400 mg every 8 h during the following 24 h, followed by 300 mg every 8 h as maintenance dose) (22 women) and ritodrine (total dose 875 mg) + placebo (22 women) in terms of PTB rate (27.2% vs. 36.4%, respectively) [75]. However, the ritodrine dose required for prolonged pregnancy was significantly less for the combination treatment than for ritodrine treatment alone (345 vs. 875 mg, *p* < 0.01) [75]. Another prospective RCT study by Arikan et al. [76] examined intravenous ritodrine alone (infusion adjustment every 20 min until maximum dose of 0.35 mg/min reached and continued until cessation of contractions or the appearance of serious maternal side effects that necessitated cessation of treatment) against ritodrine (at same infusion regimen) + vaginally administered progesterone (200 mg/d until delivery or 36 + 6 weeks gestation) in 83 women (GA, 24–34 weeks) with threatened PTL. They found no significant benefit of the combinational tocolysis compared to ritodrine alone in terms of reducing PTB rate (50% vs. 65%, respectively); however, the latency period until delivery was significantly prolonged (32 vs. 21 days, respectively) and neonatal birth weights were significantly greater (2,982.8 ± 697.8 g versus 2,585.3 ± 746.6 g, respectively) (*p* < 0.05) in the combinational tocolysis group [76].

### Beta-mimetics in Combination with Phosphodiesterase Inhibitors

#### Ex Vivo Human Studies

Franova et al. [77] examined the effects of salbutamol + rolipram in combination against OT-induced contractions in pregnant human myometrial strips. They found that salbutamol (10^− 4^ M) + rolipram (10^− 4^ M) in combination was more effective at inhibiting contraction amplitude (69.9% inhibition) than either salbutamol alone (43.93% inhibition) or rolipram alone (52.02% inhibition) at the same concentrations [77]. The study did not confirm whether this was an additive or synergistic effect, however, the reported data can be quantitatively assessed via the Bliss Independence model. Our retrospective assessment determined that in the reported experimental setting, salbutamol + rolipram in combination was additive, in that the observed contraction amplitude inhibition (69.9%) was not significantly greater or significantly less than the theoretically expected (additive) inhibition (72.7%). Verli et al. [78] examined the effects of terbutaline + rolipram in combination against OT-induced contractions in pregnant human myometrial strips. They found that the contraction-inhibitory effect of terbutaline (10^− 4^ M), assessed against contraction AUC, was potentiated by rolipram (10^− 6^ M), in that terbutaline + rolipram in combination inhibited contractions by 78.3%, which was greater than the contraction inhibition achieved by terbutaline alone (71.6%) and rolipram alone (61.9%) [78]. While the study did not determine whether this was an additive or synergistic effect, our retrospective assessment of the reported data via the Bliss Independence mode indicated that terbutaline + rolipram in combination was antagonistic, in that the observed inhibition (78.3%) was significantly less than the theoretically expected inhibition (89.1%, which was calculated from the sum of the individual tocolytic effects) at same concentrations. Moreover, when myometrial strips from PTB patients were examined, the maximum contraction (AUC) inhibition by terbutaline alone (10^− 4^ M) was 39.7%, which increased to 63.5% in combination with 10^− 6^ M of rolipram [78]. Again, based on the Bliss Independence model, the observed AUC inhibition for combination (63.5%) was significantly less than the theoretically expected inhibition (77%), indicating an antagonistic effect [78].

#### In Vivo Animal Studies

In a study of rats, Verli et al. [78] demonstrated that cumulative treatment with terbutaline (0.5–11 µg/kg) in the presence of fixed-concentration rolipram (500 µg/kg) inhibited uterine contractions (AUC) in live rats (on day 22 of pregnancy; at full term) and LPS-treated rats (on day 20 of pregnancy; at preterm), as measured by in vivo myography. The contraction inhibitory effect of terbutaline + rolipram in combination was greater than terbutaline alone (at the same concentration) for both full term and LPS-treated rats [78]. However, terbutaline + rolipram in combination primarily prevailed over terbutaline alone at low doses of terbutaline (0.5–3 µg/kg), as the effect of the combination treatment became insignificant compared to terbutaline alone at higher doses. Moreover, the IC_50_ (concentration that produced a 50% reduction in contraction AUC) of the combination treatment was not significantly different from the IC_50_ of terbutaline alone for both the full term and LPS-treated rats [78]. The authors indicated that terbutaline + rolipram in combination resulted in potentiation. Unfortunately, the data published do not allow for retrospective quantitative analyses to be applied to confirm a synergistic or additive effect.

### Beta-mimetics in Combination with Connexin-43 Inhibitors

#### Ex Vivo Human Studies

Barnett et al. demonstrated synergistic tocolysis in human myometrial strips ex vivo using a combination of 18β-glycyrrhetinic acid (10⁻⁴ M), a Cx43 inhibitor, and nebivolol (10⁻⁵ M), a beta-mimetic that is also known to induce nitric oxide production via endothelial nitric oxide synthase activation. The combination significantly reduced oxytocin-induced contraction AUC by 87.5%, compared to 18β-glycyrrhetinic acid (66.2%) and nebivolol (50.1%) alone, indicating a clear potentiation. While the original study reported that the combination effect was synergistic, our retrospective analysis, utilizing the Bliss Independence model, determined that in the described experimental setup, the combination of 18β-glycyrrhetinic acid + nebivolol was actually additive. Specifically, the observed inhibition of contraction amplitude (87.5%) was not significantly greater than the expected additive inhibition (83%) [9].

While beta-mimetics in combination with other tocolytics shows some degree of potentiation or synergism, a critical limitation of beta-mimetics exists. Beta-mimetics, such as ritodrine and salbutamol, are susceptible to β₂-adrenergic receptor (β_2_AR) desensitization when used in both monotherapy and combination regimens, following prolonged stimulation. The beta-arrestin-dependent pathway leads to this receptor desensitization, reducing β_2_AR responsiveness over time [79]. Sustained agonist exposure triggers β_2_AR phosphorylation by G protein-coupled receptor kinases, followed by beta-arrestin recruitment and internalization into endosomes. This receptor trafficking diminishes the availability of functional receptors on the myometrial cell surface, significantly attenuating tocolytic efficacy. In combination regimens, this desensitization may compromise the synergistic potential of beta-mimetics with other tocolytics, as sustained efficacy is critical for prolonged pregnancy maintenance [80]. These highlight the need for strategies that mitigate internalization, such as intermittent dosing or adjunctive agents that promote receptor recycling, to fully determine the therapeutic potential of beta-mimetics in the management of sPTL.

### Calcium Channel Blockers in Combination with Non-steroidal Anti-inflammatory Drugs

Numerous studies have examined the use of CCBs and NSAIDs for PTB prevention, however, these have primarily been as monotherapies of either CCBs or NSAIDs. Only a small number of studies have evaluated the effectiveness of combining CCBs and NSAIDs.

#### Ex Vivo Animal Studies

Doret et al. [19] examined the tocolytic effect of nicardipine (a CCB) (5.8 × 10^− 9^ M) + rofecoxib (a selective PTGS2 inhibitor, NSAID) (4.8 × 10^− 9^ M) in combination in pregnant rat myometrial strips. The combinational treatment inhibited myometrial contraction AUC (74.9%) more effectively than nicardipine alone or rofecoxib alone (50%) at the same concentrations; however, the combinational effect (74.9%) did not exceed the theoretically expected AUC inhibition of combining the drugs (75% of contraction AUC inhibition, as determined by the Bliss Independence model), indicating an additive effect [19].

#### Ex Vivo Human Studies

Kuc et al. [81] reported that nifedipine (8.68 × 10^− 9^ M) + celecoxib (a selective PTGS2 inhibitor, NSAID) (1.1 × 10^− 7^ M) in combination produced a significantly weaker tocolytic effect (∼55% contraction AUC inhibition, extracted from the original data) than the theoretically expected effect in pregnant human myometrial strips, as determined from the Bliss independence model (75% AUC inhibition, as calculated from the effects of nifedipine alone and celecoxib alone at same concentrations), thus indicating an antagonistic effect [81]. Pohl et al. [22] reported that the combination of nifedipine (6 × 10^− 9^ M) + OBE002 (a PGF_2α_ receptor antagonist) (6 × 10^− 8^ and 6 × 10^− 7^ M) resulted in a potentiation, in that the combination was significantly more effective at inhibiting OT-induced contractions in pregnant human myometrial strips than nifedipine or OBE002 alone at same concentrations. The authors inferred that the effect was synergistic, although a quantitative assessment for synergism was not performed [22]. Moreover, the data published do not allow for retrospective quantitative analyses to assess for synergistic or additive effects.

#### In Vivo Animal Studies

Pohl et al. [22] evaluated the in vivo tocolytic efficacy of nifedipine (5 mg/kg) + OBE022 (a prodrug of OBE002 and PGF_2α_ receptor antagonist) (100 mg/kg) in combination in near-term pregnant mice. Consistent with their prior ex vivo human study, the authors reported that nifedipine + OBE022 was significantly more effective at delaying RU-486-induced PTL than either agent alone at the same concentrations used for the combination treatment, indicating a significant potentiation [22]. Again, the authors inferred the effect was synergistic, although a quantitative assessment of synergism was not performed.

#### Human Clinical Studies

Kashanian et al. [18] performed an RCT on 150 women at risk of PTB with a gestational age of 26–34 weeks. Three treatment groups received either nifedipine (20 mg orally, and in case of inhibiting contraction after 2 h, 20 mg orally every 4 h for 48 h, total of 180 mg/d + rectal placebo), indomethacin (100 mg rectally, and in case of inhibiting contraction after 2 h, 25 mg orally every 4 h for 48 h, total of 200 mg/d + oral placebo), or nifedipine (oral dose as single treatment) + indomethacin (rectal dose as single treatment) in combination [18]. The study found that combinational tocolysis with nifedipine + indomethacin was more effective than either agent alone at inhibiting PTL and delaying delivery for 2 h to a maximum of 7 days. However, the study did not report definitive clinical (maternal and fetal) safety data concerning the combinational tocolysis [18].

### Calcium Channel Blockers in Combination with Oxytocin Receptor Antagonist

CCBs (mostly nifedipine) and OTRA (mostly Atosiban) are the most frequently used tocolytics for PTL. While nifedipine is still off-label in obstetrics, Atosiban is licensed and mostly used in Europe. Only a few preclinical studies have been conducted to evaluate the tocolytic efficiency of CCBs in combination with OTRAs.

#### In Vitro Studies

Siricilla et al. demonstrated the synergistic effect of mundulone, a potent inhibitor of Ca²⁺ mobilization, in combination with Atosiban. This synergistic potential was assessed through a high-throughput Ca²⁺-mobilization assay using an 8 × 8 dose matrix in human primary myometrial cells, analyzed using the Bliss Independence model. The combination of mundulone (IC_50_ = 13.25 µM) and Atosiban (IC_50_ = 3.58 µM) at a fixed dose ratio of 3.7:1 significantly enhanced both potency (resulting in a 2-fold reduction in IC_50_) and efficacy (demonstrating a 2-fold increase in E_max_ to 94.72%) compared to mundulone alone (IC_50_ > 30 µM, E_max_ = 66.3%). This combination achieved a therapeutic index (TI) of 10, a substantial improvement over mundulone alone (TI = 0.8), indicating increased safety and effectiveness [82].

#### Ex Vivo Animal Studies

Doret et al. [20] demonstrated that nicardipine (5.8 × 10^− 9^ M) + Atosiban (10^− 7^ M) in combination inhibited spontaneous contraction AUC (80%) significantly more than either of the agents alone at the same concentrations (theoretical contraction inhibition for both nicardipine and rofecoxib was 50%) in pregnant rat myometrial strips. However, the observed combinational effect (80%) was not significantly greater than the expected combinational effect (75% AUC inhibition), as determined via the Bliss Independence model, thus indicating an additive effect [20]. In another study, Siricilla et al. demonstrated that the combination of mundulone (10 pM -100 µM) and Atosiban at a fixed dose ratio of 3.7:1 significantly reduced the AUC of spontaneous contractions in myometrial strips from pregnant mice (E_max_ = 97% and IC_50_ 0.09 µM) compared to mundulone alone (E_max_ = 70% and IC_50_ = 10 µM). Additionally, when examining a single dose combination of mundulone (6.5 µM) and Atosiban (1.76 µM), the Bliss and HSA synergy scores were found to be 23.07 and 25.68, respectively. A synergy score greater than 10 indicates that the combination of the two substances exhibits synergistic effects [82].

#### Ex Vivo Human Studies

Kuc et al. [81] found that nifedipine (8.68 × 10^− 9^ M) + Atosiban (4.97 × 10^− 8^ M) in combination inhibited pregnant human myometrial contraction AUC (72.4%) significantly more than either of the agents alone (50%) at same concentrations, but did not exceed the theoretically expected effect for the tocolytic combination (75% of total contraction AUC inhibition as determined via the Bliss Independence model), once again indicating an additive effect [81]. These results are consistent with the findings of Doret et al. [20]. Furthermore, Carvajal et al. [66] demonstrated that the contraction inhibition effect of nifedipine + Atosiban in combination was greater than Atosiban alone (1 × 10^− 10^– 5 × 10^− 6^ M) but not greater than the nifedipine alone (1 × 10^− 10^– 5 × 10^− 6^ M) in pregnant human myometrial strips. The authors inferred the effect was additive, although this was not confirmed by quantitative assessment [66]. Moreover, the data published do not allow for retrospective quantitative analyses to assess for synergistic or additive effects. Siricilla et al. evaluated the tocolytic efficacy and potency of mundulone, both as a single agent and in combination with Atosiban. This evaluation was based on the AUC inhibition of KCl-induced contractions in term pregnant human myometrial tissue. Consistent with their prior ex vivo animal study, this study found that the combination of mundulone (10 pM -100 µM) + Atosiban at a fixed dose ratio of 3.7:1 demonstrated increased efficacy and potency (E_max_ = 88% and IC_50_ 0.12 µM) compared to mundulone alone (E_max_ = 57% and IC_50_ = 7 µM). Additionally, the authors reported Bliss and HSA synergy scores of 14.85 and 14.58, respectively, when testing the combination of mundulone (6.5 µM) + Atosiban (1.78 µM) in a single dose (synergy score > 10 indicates synergism) [82].

#### In Vivo Animal Studies

In a mouse model of preterm labor induced by mifepristone (30 µg) on gestational day 15, treatment with mundulone (6.5 mg/kg) and Atosiban (1.75 mg/kg), administered 5 h after induction, significantly delayed delivery. This combination therapy allowed 71% of the dams to deliver viable pups at term (gestational day 19 or later), in contrast to the single-agent treatments, which resulted in 0% for mundulone and 17% for Atosiban. Additionally, there were no viable pups reported in the mundulone group, and the average number of surviving pups born at term was not significantly different between the Atosiban and combination groups (12 vs. 9.6) [82].

#### Human Clinical Studies

In clinical settings, administering CCBs in combination with OTRAs has revealed mixed outcomes. Al-Omari et al. [83] conducted a RCT on 92 pregnant women (GA, 24–35 weeks) to compare the tocolytic efficacy and safety of nifedipine (10 mg orally every 15 min until uterine quiescence was achieved, maximum of 40 mg in the first hour, followed by a maintenance dose of 10 mg every 6 h for 48 h) + Atosiban (IV bolus dose of 6.7 mg over 1 min followed by the IV infusion of 18 mg/h for 3 h, followed by 6 mg/h for 24–48 h) in combination vs. Atosiban alone (at the same concentration as the combination treatment) for delaying delivery. There was no significant difference between the combinational tocolysis group and Atosiban alone group for delaying delivery by 48 h (91.5% vs. 91.1%, respectively) or for delaying delivery by 7 days (90.7% vs. 85.7%, respectively) [83]. In terms of tocolytic safety, neonatal outcomes were comparable with no significant difference between the combinational tocolysis and Atosiban monotherapy, however, mild cardiovascular side effects were more pronounced in women who received the combinational tocolysis (64%) compared to Atosiban alone (34%) [83]. Madkour et al. [84] conducted a prospective study of 150 women with symptoms of PTL to compare treatment with nifedipine (20 mg orally, followed by 20 mg orally after 30 min, and in case of persistent contractions, 20 mg orally every 3–8 h for 48–72 h with a maximum dose of 160 mg/d) + Atosiban (IV 6.75 mg initial dose, 300 µg/min loading dose for 3 h, 100 µg/min maintenance dose for 48–96 h) vs. nifedipine alone and Atosiban alone at same doses as used for the combination treatment. In contrast to Al-Omari et al. [83], Madkour et al. found that the combinational tocolysis group (86%) was significantly more effective at postponing labor by 7 days than nifedipine alone (60%) and Atosiban alone (56%). They also reported an increase in mild side effects associated with the combinational tocolysis therapy [84].

### Calcium Channel Blockers in Combination with Phosphodiesterase Inhibitors

#### Ex Vivo Human Study

Only one preclinical ex vivo study has examined the application of a CCB + a PDE inhibitor in combination. Chiossi et al. [85] showed that preincubating myometrial strips in sildenafil (a selective PDE5 inhibitor) (231 ng/ml, equivalent to 4.87 × 10^− 7^ M) significantly potentiated the tocolytic effect of nifedipine (10^− 10^– 10^− 5^ M) against spontaneous contractions ex vivo, shifting the concentration-response curve to the left. Moreover, the contraction inhibition by nifedipine at 10^− 8^ M in the presence of sildenafil (4.87 × 10^− 7^ M) was more pronounced (53.3%) than the nifedipine alone (24.5%) and sildenafil alone (∼15%, extracted from the original data) treatments at the same concentration [85]. The authors did not assess whether this was an additive or synergistic effect; however, our retrospective assessment of the published data using the Bliss Independence model indicated that the nifedipine + sildenafil combination was synergistic, in that the observed inhibition (53.3%) was significantly greater than the theoretically expected inhibition (35.8%, calculated from the sum of the individual drug effects) at the same concentrations.

#### Human Clinical Studies

Five recently published RCTs have shown the benefits of administering nifedipine + sildenafil in combination vs. nifedipine alone. A study by Maher et al. [86] of 239 women (GA, 24–34 weeks) with threatened PTL reported that nifedipine (initial dose, 20 mg orally, followed by 10 mg orally every 6–8 h for 48–72 h) + sildenafil (25 mg orally every 8 h for 48–72 h) (pregnancy prolongation by 29 days) was more effective at prolonging pregnancy than nifedipine alone (same dose as combination treatment) (pregnancy prolongation by 7 days). The combination tocolysis also improved neonatal outcomes, including increased neonatal birthweight and reduced NICU admissions, and was associated with fewer deliveries within 7 days of admission, compared to the nifedipine alone treatment group (delivery rate for combination was 9.1% vs. for nifedipine alone was 20.3%) [86]. A study by Qurat-ul-ain et al. [87] of 292 women (GA, 24–36 weeks) with threatened PTL reported similar findings, where 82.9% of women who received nifedipine (initial dose 20 mg orally, followed by 10 mg orally every 6–8 h for 72 h) + sildenafil (25 mg orally every 8 h for 72 h) (MGA, 31 weeks) were undelivered after 72 h of initiation of treatment, compared to 70.5% of women who received nifedipine alone (same dose as combination treatment) (MGA, 29.58 weeks). A comparative study conducted by El-Aziz et al. [88] of 96 women with threatened PTL reported that nifedipine + sildenafil in combination was more effective than nifedipine alone at delaying preterm deliveries. However, nifedipine + sildenafil in combination was associated with increased maternal cardiovascular side effects compared to nifedipine alone [88]. In similar RCT studies, Mohammadi et al. [89] and Karya et al. [90] evaluated the tocolytic efficacy of nifedipine + sildenafil in combination on 132 women and 80 women with threatened PTL, respectively. The studies reported the superiority of nifedipine (10 mg every 6–8 h, orally) + sildenafil (25 mg every 8 h, vaginally) in combination compared to nifedipine alone (same dose regimen as the combination treatment) in terms of delaying preterm deliveries for 72 h, reduced frequency of deliveries within 7 days after hospital discharge, reduced neonatal respiratory distress syndrome, and increased neonatal birthweight [89, 90].

### Calcium Channel Blockers in Combination with Progesterone

#### Ex Vivo Animal Studies

Hajagos-Tóth et al. [64] assessed the tocolytic effect of cumulative doses of nifedipine (10^− 11^– 10^− 6^ M) against KCl-stimulated contractions in rat uterine tissue that was pre-treated with progesterone (0.5 mg in 0.1 mL of corn oil) from fetal gestation days 15–21. They found that nifedipine abolished contractions in a dose-dependent manner without progesterone pre-treatment, but interestingly, the tocolytic effect of nifedipine was significantly impeded in the strips from animals pre-treated with progesterone, indicating an antagonistic effect. However, antagonism was not confirmed via the application of an assessment model [64].

#### Ex Vivo Human Studies

Baumbach et al. [91]. compared the tocolytic effect of nifedipine with or without progesterone using pregnant human myometrial strips. The study showed that a single dose of nifedipine (10^− 8^ M) and progesterone (10^− 5^ M) in separate strips reduced spontaneous contractility (AUC) by ∼40% and ∼5%, respectively, within 1 h. However, the degree of contraction inhibition was significantly more pronounced (∼65%) in strips pre-incubated with progesterone and then treated with nifedipine [91], which contrasts the findings of the ex vivo study by Hajagos-Tóth et al. [64] on rat myometrium. It was unclear whether the effect was additive or synergistic; however, the authors inferred that progesterone either additively or synergistically potentiated the contraction inhibitory effect of nifedipine. Our retrospective assessment of the reported data using the Bliss Independence model has confirmed that nifedipine + progesterone in combination was synergistic, in that the observed inhibition (∼65%) was significantly greater than the theoretically expected inhibition (∼43%).

#### In Vivo Animal Studies

In a rat model of hormone-induced preterm labor, Hajagos-Tóth et al. [67] compared the tocolytic efficacy of nifedipine (3.89 mg/d per animal) + progesterone (0.5 mg/d) in combination vs. nifedipine alone. Nifedipine was administered on fetal gestation days 16 and 18 to pregnant rats that had or had not received progesterone from fetal gestation day 15 until delivery [67]. Animals that received nifedipine + progesterone were less likely to have a delayed birth than the animals that received nifedipine alone [67]. The authors concluded that progesterone impeded the effect of nifedipine in the combination treatment, indicative of an antagonistic effect, which is consistent with findings from their ex vivo rat study [64].

#### Human Clinical Studies

Areeruk et al. [92] performed a randomized, placebo-controlled, double-blind study to compare nifedipine (10 - 20 mg orally every 6 h) + progesterone (20 mg/d of oral dydrogesterone until delivery or until the GA, 37 weeks) in combination vs. nifedipine (same dose regimen as the combination) + placebo. In the study, 48 pregnant women (GA, 24–34 weeks) received nifedipine; of those, 24 women received additional dydrogesterone treatment while 24 women received an additional placebo [92]. There were no differences found between the nifedipine + progesterone and nifedipine + placebo treatment groups for the primary outcomes assessed, which were recurrent uterine contraction rate (87.5% vs. 91.7%, respectively) and pregnancy latency period (32.7 days for combination treatment vs. 38.2 days for nifedipine treatment) [92]. There were also no significant differences between assessed secondary outcomes of gestational age at delivery, pregnancy outcomes, and neonatal outcomes [92]. However, two RCT studies by Nisa et al. [93] and El-Sayed et al. [94] reported significant benefits of nifedipine + progesterone in combination for acute tocolysis. The studies were conducted on 60 and 52 women with threatened PTL, respectively. They reported that combinational tocolysis with nifedipine + progesterone was more effective than nifedipine alone (same dose regimen as combination treatment) in terms of stoppage of uterine contractions [93] and rapid response to tocolysis [94], while having similar feto-maternal side effects [94].

### Calcium Channel Blockers in Combination with Other Calcium Channel Blockers

#### In Vitro Studies

In a study by Siricilla et al., the combination of mundolene (IC_50_, 6.82 µM) and nifedipine (IC_50_, > 30 µM) at a fixed dose ratio of 1:44 increased both potency (3-fold reduction in IC_50_) and efficacy (6-fold increase in E_max_, 67.63%) compared to mundolene (IC_50_, 18.22 µM, E_max_, 11.08%) alone. This synergistic potential was evaluated using a high-throughput Ca²⁺-mobilization assay to measure the intracellular calcium transients with an 8 × 8 dose matrix in human primary myometrial cells, analyzed through the Bliss Independence model [82].

### Non-steroidal Anti-inflammatory Drugs in Combination with Oxytocin Receptor Antagonists

#### Ex Vivo Animal Studies

Doret et al. [20]. assessed the tocolytic effects of rofecoxib (4.8 × 10^− 9^ M) + Atosiban (10^− 7^ M) in combination on contracting pregnant rat myometrial strips ex vivo. The study reported that rofecoxib + Atosiban more effectively inhibited myometrial contractions than either agent alone (50%) at the same concentration, but the effect size (73.5%) was not significantly greater than the theoretical expected additive effect (75% contraction AUC inhibition, as determined via the Bliss Independence model), indicating an additive effect [20].

#### Ex Vivo Human Studies

Kuc et al. [81] reported that celecoxib (5.71 × 10^− 9^ M) + Atosiban (4.97 × 10^− 8^ M) inhibited myometrial contractility (∼65% AUC inhibition, extracted from the original data) significantly less than the theoretically expected additive effect (75% contraction inhibition, as determined via the Bliss Independence model), in pregnant human myometrial strips. This indicates an antagonistic effect [81]. Pohl et al. [22] evaluated the tocolytic effect of Atosiban (6 × 10^− 9^ M) in combination with OBE002 (a PGF_2α_ receptor antagonist) (6 × 10^− 8^ and 6 × 10^− 7^ M) in pregnant human myometrial strips. Co-administration of OBE002 potentiated the contraction inhibitory effect of Atosiban, indicating a potentiation effect [22]. However, the published data do not allow for retrospective quantitative analyses to confirm either a synergistic or additive effect.

### Non-steroidal Anti-inflammatory Drugs in Combination with Progesterone

#### Ex Vivo Human Study

Baumbach et al. [91]. showed that myometrial strips treated with a single dose of indomethacin (10^− 6^ M) and progesterone (10^− 5^ M) in separate strips reduced spontaneous contraction AUC by ∼10% and ∼5%, respectively, within 1 h. The contraction inhibition was significantly more pronounced (∼30% ) in strips that had been pre-incubated with progesterone and then treated with indomethacin [91]. It was unclear whether the effect was additive or synergistic; however, the authors inferred that progesterone either additively or synergistically potentiated the contraction inhibitory effect of indomethacin [91]. Nevertheless, our retrospective quantitative assessment of the reported data via the Bliss Independence model indicated that the indomethacin + progesterone combination was synergistic, in that the observed inhibition (∼30%) was significantly greater than the theoretically expected additive inhibition (∼15%).

### Oxytocin Receptor Antagonists in Combination with Magnesium Sulfate

#### Ex Vivo Human Study

Arrowsmith et al. [95] compared the tocolytic potency of magnesium sulfate (2 × 10^− 3^– 1.2 × 10^− 2^ M) with or without Atosiban (10^− 7^ M) against the OT-induced myometrial contractions. The study reported that the tocolytic potency of magnesium sulfate was significantly higher in the presence of Atosiban, compared to magnesium sulfate alone [95]. The concentration-response analysis revealed that the combination treatment significantly shifted the concentration-response curve to the left and, consequently, the IC_50_ (concentration that produced a 50% reduction in contraction AUC) for magnesium sulfate in the combination was significantly lower than the IC_50_ for magnesium sulfate alone (2.81 vs. 4.74 mM, respectively), indicating a significant potentiation. However, the potentiation effect was not confirmed via the application of a model for assessing synergism.

### Progesterone in Combination with Magnesium Sulfate

#### Ex Vivo Human Study

Baumbach et al. [91]. also compared the tocolytic effect of magnesium sulfate with or without progesterone. The study showed that magnesium sulfate inhibited spontaneous contractions in a concentration-dependent manner (2 × 10^− 3^, 4 × 10^− 3^ and 8 × 10^− 3^ M) and that co-treatment with progesterone (10^− 5^ M) made no significant difference to the contraction inhibitory effect of the magnesium sulfate [91]. The published data do not allow for retrospective quantitative analyses to confirm an antagonistic, additive, or synergistic effect.

### Progesterone in Combination with Phosphodiesterase Inhibitors

#### Ex Vivo Human Study

Lai et al. [21]. evaluated the tocolytic effect of progesterone (10^− 7^ M and 3 × 10^− 7^ M) + aminophylline (a nonselective PDE inhibitor) (2.5 and 7.5 × 10^− 4^ M) in combination against OT-induced contractions in pregnant human myometrial strips. The contraction inhibitory effect of progesterone + aminophylline was not significantly greater than either of the agents alone at the same concentrations used in the combination treatment [21]. The published data do not allow for retrospective quantitative analyses.

#### In Vivo Animal Study

In an LPS-induced mouse model of PTL, Herbert et al. [23] reported that the combination of progesterone (5 mg/kg) + aminophylline (10 mg/kg) prolonged pregnancy by 74 h, which was significantly longer than the prolongation achieved by progesterone alone (11.6 h, *p* < 0.01) and aminophylline alone (12.9 h, *p* < 0.01) at the same concentrations, indicating a significant potentiation effect for the combination treatment. However, the potentiation effect was not confirmed via the application of an assessment model. Moreover, the survival rates of live pups were not improved with progesterone alone, aminophylline alone, or their combination [23].

### Other Tocolytic Combinations

#### Ex Vivo Human Studies

Hyuga et al. [96] evaluated the tocolytic potency of two anoctamin 1 antagonists, benzbromarone and MONNA, combined with nifedipine or terbutaline in human myometrial strips. The study reported that the combinations of nifedipine or terbutaline with benzbromarone or MONNA were synergistic, in that the combination treatments reduced OT-induced myometrial contractions to a significantly greater degree than the respective single agents alone at the same concentrations [96]. However, there was no quantitative assessment performed to confirm synergism [96]. Santos et al. [97] evaluated the tocolytic effect of nifedipine or Atosiban in the presence of *Bryophyllum pinnatum* plant juice against contracting pregnant human myometrial strips. *Bryophyllum pinnatum* is an herbal medicine that has been used as a tocolytic agent in anthroposophic medicine and, conventionally, it is used alone or as a co-treatment with tocolytic agents, such as Atosiban or nifedipine. The results showed that *Bryophyllum pinnatum* (2.5 µg/mL*)* potentiated the contraction inhibitory effect of both nifedipine (3 ng/mL) and Atosiban (0.27 µg/mL) against spontaneous pregnant human myometrial contractions, suggesting a synergistic effect [97]. However, no quantitative assessment was performed to confirm synergism [97]. The active ingredient of *Bryophyllum pinnatum* juice and the mechanism(s) by which it potentiates the inhibitory effects of nifedipine and Atosiban are currently unknown.

#### In Vivo Animal Study

In an LPS-induced mouse model of PTB, Zierden et al. [98] reported that the vaginal administration of progesterone (1 mg) + Trichostatin A (a potent histone deacetylase inhibitor) (15 µg) was significantly more effective at reducing rates of LPS-induced PTB (PTB rate, 60% vs. 90% for control group, *p* = 0.003) than the same concentrations of either progesterone alone (PTB rate, 85% vs. 90% for control group) and Trichostatin A alone (PTB rate, 70% vs. 90% for control group). However, the study did not confirm either synergism or additive effect through quantitative assessment. Nonetheless, progesterone + Trichostatin A in combination resulted in the delivery of live offspring that exhibited neurotypical development [98].

Tocolytic combinations that have been examined in preclinical settings are listed in Table 1. Table 2 lists the preclinical combinational studies for which we have, in the present review, conducted retrospective quantitative assessment for tocolytic synergism, additivity, or antagonism, by applying the Bliss Independence model to the published data. In doing so, we have confirmed numerous instances of tocolytic synergism within the existing literature. Table 3 shows the list of tocolytic combinations trailed in clinical settings.

## Summary

Evidence suggests that PTL is a syndrome attributable to multiple pathological processes, including inflammation, with or without infection, uterine overdistension, maternal stress, ischemia or hemorrhage, endocrine disorders, immunologically mediated processes [16], and a gene expression pattern distinct from term labor [99]. Thus, the management of PTL is complicated by the need to address multiple dysfunctions simultaneously.

The concept of combinational tocolysis encompasses several classes of drugs acting simultaneously upon distinct biochemical signaling pathways within uterine myocytes. In contrast, monotherapy tocolysis is typically limited to targeting only one aspect of myometrial pro-contractile signaling and, as such, may be insufficient to address the multifactorial drivers underpinning PTL. It is unsurprising, therefore, that monotherapy approaches have proven to have limited efficacy while compromising neonatal safety due to dose-related side effects. Given these caveats, combining two or more tocolytics that target different myocyte signaling pathways may pave a beneficial pathway toward effective tocolysis by synergisms conferring therapeutic efficacy at lower drug doses, thereby reducing side effects. Accordingly, prior studies (across ex vivo, in vivo, and human RCTs) have assessed the benefits of combining a range of different tocolytics. Many synergistic tocolytic combinations have been identified in preclinical settings or entered the clinical stage for further investigation; however, there are some conflicting reports in the literature. Some studies show no benefit to combinational tocolysis; for example, nifedipine + Atosiban, nifedipine + progesterone, and progesterone + magnesium sulfate combinations have shown no significant improvement to contraction inhibition in preclinical studies [19–22, 66, 81], while in the clinical setting, ritodrine + magnesium sulfate and nifedipine + progesterone combinations have failed to show clinical benefits in terms of reduced PTB rate or pregnancy prolongation [71, 72, 75, 76, 100–102]. However, other preclinical studies have shown that combinations of ritodrine + nifedipine, rolipram + salbutamol, nifedipine + sildenafil, and Atosiban + magnesium sulfate all showed either additive or synergistic effect [19, 20, 22, 23, 63–67, 74, 77, 78, 85, 91, 95, 96]. Moreover, in clinical settings, some combinations, such as nifedipine + indomethacin, nifedipine + sildenafil, and nifedipine + salbutamol, have shown clear clinical benefits in terms of reduced PTB rate, pregnancy prolongation, reduced maternal side effects, reduced NICU admission, reduced hospital stay, and improved neonatal wellbeing [18, 68, 69, 76, 84, 86, 87, 89, 90, 103–107]. Combinational tocolysis may therefore have great potential for improving PTB management; however, the conflicting outcomes indicate that scrutiny is required when selecting which tocolytics to combine. Notwithstanding the preceding caveat, it remains possible that combinational tocolysis may at least provide tocolytic redundancy, helping to ensure patients meaningfully respond to at least one of the administered tocolytics [24]. If tocolytic synergism can be achieved in vivo, this at least raises the prospect of safer acute tocolysis by reducing the feto-maternal side effects, and maybe even maintenance tocolysis.

However, despite revealing some promising benefits, including within clinical trial settings (Table 3), combinational tocolysis has yet to be translated into routine clinical practice. The limited clinical adoption stems from a combination of modest clinical benefits, heterogeneous study designs, safety concerns, insufficient large-scale trials, translational challenges, regulatory barriers, and unclear differentiation of additive versus synergistic effects. To overcome these barriers, future research should focus on conducting large, well-designed clinical trials with standardized outcome measures, clear safety profiles, and robust analyses of synergistic effects to establish the efficacy and practicality of combinational tocolysis for routine use in preventing preterm birth.

### Future Perspectives

As studies into tocolytic synergism continue to progress, implementation of robust quantitative assessment is required, incorporating a well-established mathematical approach or suitable reference model. Current trends toward investigating tocolytic combinations in obstetrics, especially in the preclinical settings, do not include a thorough quantitative assessment. Rather, most investigations are limited to comparing the combination effect vs. the single effect of the constituent drugs, which does not constitute an assessment of synergism. In the present review, we have been able to perform retrospective quantitative assessment for synergism for multiple prior studies, but for many studies, the reported data do not permit such retrospective quantitation. Going forward, the effects of tocolytic combinations should be quantified with a suitable reference model and mathematical approach to provide evidence of potentiation/synergism, compared to the single agents [46, 61]. Additionally, multiple studies examined in this review implemented a sequential therapeutic approach [64, 67, 78, 85]; this presents its own unique challenges for quantitation, such as determining the importance of timing between the administration of the different tocolytics [37]. Moreover, a primary objective of combinational tocolysis is achieving the cessation of PTL with reduced tocolytic concentrations; unfortunately, however, most preclinical studies have not assessed whether tocolytic combinations allow contraction inhibition to be achieved with reduced tocolytic concentrations. Hence, future combinational tocolysis studies should aim to demonstrate whether a tocolytic combination can achieve the abolition of myometrial contractions at reduced concentrations/doses, compared to concentrations/doses required for the individual constituent agents.

Furthermore, as previously mentioned, some tocolytics combinations are reported to improve PTB management whereas others do not. Currently, there is no formula for predicting whether combining two or more tocolytics will improve PTB management, have no benefit, or even worsen outcomes. Furthermore, the pregnant human uterus lacks significant sympathetic nervous innervation, unlike vascular or gastrointestinal smooth muscle, which may limit the efficacy of tocolytics repurposed from other smooth muscle contexts (e.g., terbutaline for asthma). Additionally, the absence of a definitive pacemaker in the myometrium complicates the targeting of contraction initiation. The lack of a pacemaker, combined with altered gap junction activity (e.g., via Cx43) and calcium signaling in sPTL, necessitates tocolytics tailored to uterine-specific pathways. As combinational tocolysis studies continue to expand, cross-referencing current and future reported clinical benefits vs. tocolytic mechanism(s) of action may one day allow us to predict which tocolytic combinations will lead to clinical benefits, based on the contraction signaling pathways being targeted.

Lastly, in treating PTB by combinational tocolysis, questions remain open regarding the safety and efficacy of tocolytics if delivered without a targeted delivery system. Therefore, progress may come through integrating combinational tocolysis with advanced drug delivery strategies, including uterine-targeted nanoliposomes [108, 109] or vaginally administered mucus-penetrating nanoparticles [110]. Such an approach has the potential to functionally regulate the release profiles of multiple drugs from a single formulation within the uterus [111]. As such, a “*uterine-targeted combinational tocolysis*” approach could potentially change how we utilize existing tocolytics and may represent a pathway toward safer, more effective tocolysis.

**Table 1:** Tocolytic combinations that have been examined in preclinical studies

Tocolytic groups	Tocolytic combination	Experimental model	Changes in myometrial contraction or pregnancy prolongation	Combination remarks and author/year
Betamimetics + Calcium channel blockers	Ritodrine + Nifedipine	Ex vivo pregnant rat myometrial strips	Significant inhibition by combination compared to the single agent alone	Synergistic *Gallagher et al. 1992* [63]
		Ex vivo pregnant human myometrial strips	Significant inhibition by combination compared to the single agent alone	Potentiation *Carvajal et al. 2017* [66]
		Ex vivo pregnant human myometrial strips	Significant inhibition by combination compared to the single agent alone	Potentiation *Saade et al. 1994* [65]
	Ritodrine + Nicardipine	Ex vivo pregnant rat myometrial strips	Observed contraction inhibition was not significantly greater than the theoretically expected inhibition	Additive *Doret et al. 2003* [20]
	Terbutaline + Nifedipine	Ex vivo pregnant rat and human myometrial strips	Significant inhibition by combination compared to the single agent alone	Potentiation *Hajagos-Tóth et al. 2009* [64] *Hajagos-Tóth et al. 2010* [67]
	Salmeterol + Nifedipine	In vivo pregnant rat	Significant pregnancy prolongation by combination compared to the single agent alone	Potentiation *Hajagos-Tóth et al. 2010* [67]
Betamimetics + Non-steroidal anti- inflammatory drugs	Ritodrine + Rofecoxib	Ex vivo pregnant rat myometrial strips	Observed contraction inhibition was significantly greater than the theoretically expected inhibition	Synergistic *Doret et al. 2002* [19]
Betamimetics + Magnesium sulphate	Ritodrine + Magnesium sulphate	Ex vivo pregnant human myometrial strips	Significant inhibition by combination compared to the single agent alone	Potentiation *Saade et al. 1994* [64]
	Terbutaline + Magnesium sulphate	Ex vivo pregnant human myometrial strips	Significant inhibition by combination compared to the single agent alone	Potentiation *Saade et al. 1994* [65]
Betamimetics + Progesterone	Salmeterol + Progesterone	In vivo pregnant rat	Significant pregnancy prolongation by combination compared to the single agent alone	Potentiation *Galik et al. 2008* [74]
Betamimetics + Phosphodi-esterase inhibitors	Salbutamol + Rolipram	Ex vivo pregnant human myometrial strips	Significant inhibition by combination compared to the single agent alone	Additive *Franova et al. 2009* [77]
	Terbutaline + Rolipram	Ex vivo pregnant human myometrial strips	Significant inhibition by combination compared to the single agent alone	Antagonistic *Verli et al. 2013* [78]
	Terbutaline + Rolipram	In vivo pregnant rat	Significant contraction inhibition by combination compared to the single agent alone	Potentiation *Verli et al. 2013* [78]
Betamimetics + Cx43 inhibitor	18β-glycyrrhetinic acid + Nebivolol	Ex vivo pregnant human myometrial strips	Significant inhibition by combination compared to the single agent alone	Synergistic *Barnett et al. 2021* [9]
Calcium channel blockers + Non-steroidal anti- inflammatory drugs	Nicardipine + Rofecoxib	Ex vivo pregnant rat myometrial strips	Observed contraction inhibition was not significantly greater than the theoretically expected inhibition	Additive *Doret et al. 2002* [19]
	Nifedipine + OBE002	Ex vivo pregnant human myometrial strips	Significant inhibition by combination compared to the single agent alone	Potentiation *Pohl et al. 2018* [22]
	Nifedipine + Celecoxib	Ex vivo pregnant human myometrial strips	Observed contraction inhibition was significantly less than the theoretically expected inhibition	Antagonistic *Kuc et al. 2011* [81]
	Nifedipine + OBE022	In vivo pregnant mouse	Significant pregnancy prolongation by combination compared to the single agent alone	Potentiation *Pohl et al. 2018* [22]
Calcium channel blockers + Oxytocin receptor antagonist	Nicardipine + Atosiban	Ex vivo pregnant rat myometrial strips	Observed contraction inhibition was not significantly greater than the theoretically expected inhibition	Additive *Doret et al. 2003* [20]
	Nifedipine + Atosiban	Ex vivo pregnant human myometrial strips	Observed contraction inhibition was not significantly greater than the theoretically expected inhibition	Additive *Kuc et al. 2011* [81]
	Nifedipine + Atosiban	Ex vivo pregnant human myometrial strips	No significant inhibition by combination compared to the single agent alone	Additive *Carvajal et al. 2017* [66]
	Mundolone + Atosiban	In vitro Ca^2+^ mobilization assay	Observed inhibition of of intracellular Ca^2+^ release was significantly greater than the theoretically expected inhibition	Synergistic *Siricilla et al. 2023* [82]
	Mundolone + Atosiban	Ex vivo pregnant mouse myometrial strips	Observed contraction inhibition was not significantly greater than the theoretically expected inhibition	Synergistic *Siricilla et al. 2023* [82]
	Mundolone + Atosiban	Ex vivo pregnant human myometrial strips	Observed contraction inhibition was not significantly greater than the theoretically expected inhibition	Synergistic *Siricilla et al. 2023* [82]
	Mundolone + Atosiban	In vivo pregnant mouse	Significant pregnancy prolongation by combination compared to the single agent alone	Synergistic *Siricilla et al. 2023* [82]
Calcium channel blockers + Phosphodi-esterase inhibitors	Nifedipine + Sildenafil	Ex vivo pregnant human myometrial strips	Significant inhibition by combination compared to the single agent alone No claim	Synergistic *Chiossi et al. 2010* [85]
Calcium channel blockers + Progesterone	Nifedipine + Progesterone	Ex vivo pregnant rat myometrial strips	Contraction inhibition by nifedipine was impeded in presence of Progesterone No claim	Antagonistic *Hajagos-Tóth et al. 2009* [64]
	Nifedipine + Progesterone	Ex vivo pregnant human myometrial strips	Contraction inhibition by nifedipine was potentiated in presence of progesterone	Synergistic *Baumbach et al. 2012* [91]
	Nifedipine + Progesterone	In vivo pregnant rat	Pregnancy prolongation was more pronounced in nifedipine treatment compared to combination treatment	Antagonistic *Hajagos-Tóth et al. 2009* [64]
Calcium channel blockers + Calcium channel blockers	Nifedipine + Mundolone	In vitro Ca^2+^ mobilization assay	Observed inhibition of of intracellular Ca^2+^ release was significantly greater than the theoretically expected inhibition	Synergistic *Siricilla et al. 2023* [82]
Non-steroidal anti- inflammatory drugs + Oxytocin receptor antagonist	Rofecoxib + Atosiban	Ex vivo pregnant rat myometrial strips	Observed contraction inhibition was not significantly greater than the theoretically expected inhibition	Additive *Doret et al. 2002* [19]
	Celecoxib + Atosiban	Ex vivo pregnant human myometrial strips	Observed contraction inhibition was significantly less than the theoretically expected inhibition	Antagonistic *Kuc et al. 2011* [81]
	OBE002 + Atosiban	Ex vivo pregnant human myometrial strips	No significant inhibition by combination compared to the single agent alone	Potentiation *Pohl et al. 2018* [22]
Non-steroidal anti- inflammatory drugs + Progesterone	Indomethacin + Progesterone	Ex vivo pregnant human myometrial strips	Contraction inhibition by indomethacin was potentiated in presence of progesterone	Synergistic *Baumbach et al. 2012* [91]
Oxytocin receptor antagonist + Magnesium sulphate	Atosiban + Magnesium sulphate	Ex vivo pregnant human myometrial strips	Contraction inhibition by magnesium sulphate was potentiated in presence of Atosiban	Potentiation *Arrowsmith et al. 2016* [95]
Progesterone + Magnesium sulphate	Progesterone + Magnesium sulphate	Ex vivo pregnant human myometrial strips	Contraction inhibition by magnesium sulphate was not potentiated in presence of progesterone	*Baumbach et al. 2012* [91]
Progesterone + Phosphodi-esterase inhibitors	Progesterone + Aminophylline	Ex vivo pregnant human myometrial strips	No significant contraction inhibition by combination compared to single agent alone	*Lai et al. 2021* [21]
	Progesterone + Aminophylline	In vivo pregnant mouse	Significant pregnancy prolongation by combination compared to the single agent alone	Potentiation *Herbert et al. 2019* [23]
Miscellaneous tocolytic combinations	Nifedipine + Benzbromarone or MONNA	Ex vivo pregnant human myometrial strips	Significant inhibition by combination compared to the single agent alone	Potentiation *Hyuga et al. 2021* [96]
	Terbutaline + Benzbromarone or MONNA	Ex vivo pregnant human myometrial strips	Significant inhibition by combination compared to the single agent alone	Potentiation *Hyuga et al. 2021* [96]
	*Bryophyllum pinnatum* plant juice + Nifedipine or Atosiban	Ex vivo pregnant human myometrial strips	Contraction inhibition by Nifedipine and Atosiban was potentiated in presence of *Bryophyllum pinnatum* plant juice	Potentiation *Santos et al. 2019* [97]

**Table 2:** List of preclinical combinational studies that have been retrospectively assessed for synergism, additivity, or antagonism in the present review. Published data were extracted and then quantitatively assessed via the bliss independence model

Tocolytic combination	Experimental model and author/year	Original observation on myometrial contraction	Revised observation on myometrial contraction based on the quantitative assessment
Ritodrine + Nifedipine	Ex vivo pregnant rat myometrial strips *Gallagher et al. 1992* [63]	Significant inhibition by combination compared to the single agent alone *(No inference)*	Observed contraction inhibition is greater than the theoretically expected inhibition *(Synergistic)*
Ritodrine + Nicardipine	Ex vivo pregnant rat myometrial strips *Doret et al. 2003* [20]	Observed contraction inhibition was not significantly greater than the theoretically expected inhibition *(Additive)*	Observed contraction inhibition is not significantly greater than the theoretically expected inhibition *(Additive)*
Ritodrine + Rofecoxib	Ex vivo pregnant rat myometrial strips *Doret et al. 2002* [19]	Observed contraction inhibition was significantly greater than the theoretically expected inhibition *(Synergistic)*	Observed contraction inhibition is significantly greater than the theoretically expected inhibition *(Synergistic)*
Salbutamol + Rolipram	Ex vivo pregnant human myometrial strips *Franova et al. 2009* [77]	Significant inhibition by combination compared to the single agent alone *(No inference)*	Observed contraction inhibition is not significantly greater than the theoretically expected inhibition *(Additive)*
Terbutaline + Rolipram	Ex vivo pregnant human myometrial strips *Verli et al. 2013* [78]	Significant inhibition by combination compared to the single agent alone *(Potentiation)*	Observed contraction inhibition is significantly less than the theoretically expected inhibition *(Antagonistic)*
18β-glycyrrhetinic acid + Nebivolol	Ex vivo pregnant human myometrial strips *Barnett et al. 2021* [9]	Significant inhibition by combination compared to the single agent alone *(Synergistic)*	Observed contraction inhibition is not significantly greater than the theoretically expected inhibition *(Additive)*
Nicardipine + Rofecoxib	Ex vivo pregnant rat myometrial strips *Doret et al. 2002* [19]	Observed contraction inhibition was not significantly greater than the theoretically expected inhibition *(Additive)*	Observed contraction inhibition is not significantly greater than the theoretically expected inhibition *(Additive)*
Nifedipine + Celecoxib	Ex vivo pregnant human myometrial strips *Kuc et al. 2011* [81]	Observed contraction inhibition was significantly less than the theoretically expected inhibition *(Antagonistic)*	Observed contraction inhibition is significantly less than the theoretically expected inhibition *(Antagonistic)*
Nicardipine + Atosiban	Ex vivo pregnant rat myometrial strips *Doret et al. 2003* [20]	Observed contraction inhibition was not significantly greater than the theoretically expected inhibition *(Additive)*	Observed contraction inhibition is not significantly greater than the theoretically expected inhibition *(Additive)*
Nifedipine + Atosiban	Ex vivo pregnant human myometrial strips *Kuc et al. 2011* [81]	Observed contraction inhibition was not significantly greater than the theoretically expected inhibition *(Additive)*	Observed contraction inhibition is not significantly greater than the theoretically expected inhibition *(Additive)*
Nifedipine + Sildenafil	Ex vivo pregnant human myometrial strips *Chiossi et al. 2010* [85]	Significant inhibition by combination compared to the single agent alone *(Potentiation)*	Observed contraction inhibition is significantly greater than the theoretically expected inhibition *(Synergistic)*
Nifedipine + Progesterone	Ex vivo pregnant human myometrial strips *Baumbach et al. 2012* [91]	Contraction inhibition by nifedipine was potentiated in presence of progesterone *(Additive or synergistic)*	Observed contraction inhibition is significantly greater than the theoretically expected inhibition *(Synergistic)*
Rofecoxib + Atosiban	Ex vivo pregnant rat myometrial strips *Doret et al. 2002* [19]	Observed contraction inhibition was not significantly greater than the theoretically expected inhibition *(Additive)*	Observed contraction inhibition is not significantly greater than the theoretically expected inhibition *(Additive)*
Celecoxib + Atosiban	Ex vivo pregnant human myometrial strips *Kuc et al. 2011* [81]	Observed contraction inhibition was significantly less than the theoretically expected inhibition *(Antagonistic)*	Observed contraction inhibition is significantly less than the theoretically expected inhibition *(Antagonistic)*
Indomethacin + Progesterone	Ex vivo pregnant human myometrial strips *Baumbach et al. 2012* [91]	Contraction inhibition by nifedipine was potentiated in presence of progesterone *(Additive or synergistic)*	Observed contraction inhibition is significantly greater than the theoretically expected inhibition *(Synergistic)*

**Table 3:** Tocolytic combinations trailed in clinical settings

Tocolytic groups	Tocolytic combination	Total = *n* (combination vs. single treatment)	Clinical Findings (combination vs. single treatment)		Comment and author/year
			PTB rate	Pregnancy prolongation	
Betamimetics + Calcium channel blockers	Ritodrine + Nifedipine (Combination vs. ritodrine)	Total = 213 (104 vs. 109)	No information	7 days delay (59.6% vs. 72.1%) (*p* = 0.05)	Combination was more effective than Ritodrine alone. *Kim et al. 2014* [68]
	Salbutamol + Nifedipine (Combination vs. Nifedipine)	Total = 76 (38 vs. 38)	*(< 37 weeks)* 10.5% vs. 39.5%	5.21 weeks vs. 6.74 weeks (*p* < 0.05)	Nifedipine alone was more effective than combination. *Qiu et al. 2015* [69]
Betamimetics + Non-steroidal anti- inflammatory drugs	Ritodrine + Indomethacin (Combination vs. Ritodrine)	Total = 44 (22 vs. 22)	*(< 37 weeks)* 31.8% vs. 63.6% (*p* < 0.05)	41.3 days vs. 25.9 days (*p* < 0.05)	Combination was more effective than Ritodrine alone. *Gamissans et al. 1978* [70]
Betamimetics + Magnesium sulphate	Ritodrine + Magnesium sulphate (Combination vs. Magnesium sulphate )	Total = 128 (64 vs. 64)	No information	28.3 h vs. 21.5 h (*p* < 0.05)	Combination was less effective than Magnesium sulphate alone. *Shuifang et al. 2020* [71]
	Ritodrine + Magnesium sulphate (Combination vs. Ritodrine)	Total = 50 (28 vs. 22)	No information	11.5 days vs. 16.8 days (*p* > 0.05)	Combination was less effective than Magnesium sulphate alone. *Ferguson et al. 1984* [72]
Betamimetics + Progesterone	Progesterone + Ritodrine (Combination vs. ritodrine)	Total = 44 (22 vs. 22)	*(< 37 weeks)* 27.2% vs. 36.4% (*p* > 0.05)	19 days vs. 21 days (*p* > 0.05)	Combination was as effective as Ritodrine alone. *Noblot et al. 1991* [75]
	Progesterone + Ritodrine (Combination vs. Ritodrine)	Total = 83 (43 vs. 40)	*(< 37 weeks)* 50% vs. 65% (*p* > 0.05)	32 days vs. 21 days (*p* > 0.05)	Combination was more effective than Ritodrine alone. *Arikan et al. 2011* [76]
Calcium channel blockers + Non-steroidal anti- inflammatory drugs	Nifedipine + Indomethacin vs. Nifedipine or Indomethacin alone	Total = 150 Combination (50) vs. Nifedipine (50) or Indomethacin alone (50)	*(< 37 weeks)* Combination (41.5%) vs. Nifedipine (75%) (*p* < 0.05) or Indomethacin alone (75%) (*p* < 0.05)	*48 h delay* Combination (95.1%) vs. Nifedipine (86.1%) or Indomethacin alone (83.3%) (*p* < 0.05) *7 days delay* Combination (90.2%) vs. Nifedipine (77.7%) or Indomethacin alone (72.2%) (*p* < 0.05)	Combination was more effective than Nifedipine or Indomethacin alone. *Kashanian et al. 2019* [18]
Calcium channel blockers + Oxytocin Receptor Antagonist	Nifedipine + Atosiban (Combination vs. Atosiban)	Total = 92 (45 vs. 47)	No information	*48 h delay* 91.5% vs. 91.1% (*p* > 0.05) 7 days delay 90.7% vs. 85.7% (*p* > 0.05)	Combination treatment was as effective as Atosiban alone. *Al-Omari et al. 2013* [83]
	Nifedipine + Atosiban vs. Nifedipine or Atosiban alone	Total = 150 Combination (50) vs. Nifedipine (50) or Indomethacin alone (50)	No information	*7 days delay* Combination (86%) vs. Nifedipine (60%) or Indomethacin alone (56%) (*p* < 0.05)	Combination treatment was more effective than Nifedipine or Atosiban alone. *Madkour et al. 2013* [84]
Calcium channel blockers + Phosphodi-esterase inhibitors	Nifedipine + Sildenafil (Combination vs. Nifedipine)	Total = 239 (121 vs. 118)	*28 to < 32 weeks* (20.7% vs. 38.1%) (*p* < 0.05) *32 to < 37 weeks* (46.3% vs. 37.3%) (*p* > 0.05)	29 days vs. 7 days (*p* < 0.05)	Combination treatment was more effective than Nifedipine alone. *Maher et al. 2018* [86]
	Nifedipine + Sildenafil (Combination vs. Nifedipine)	Total = 292 (146 vs. 146)	No information	*72 h delay* 82.9% vs. 70.5% (*p* < 0.05)	Combination treatment was more effective than Nifedipine alone. *Qurat-ul-ain et al. 2020* [87]
	Nifedipine + Sildenafil (Combination vs. Nifedipine)	Total = 139 (70 vs. 69)	No information	16.17 days vs. 9.98 days (*p* < 0.05)	Combination treatment was more effective than Nifedipine alone. *Mohammadi et al. 2021* [89]
	Nifedipine + Sildenafil (Combination vs. Nifedipine)	Total = 80 (40 vs. 40)	No information	28.23 days vs. 12.98 days (*p* < 0.05)	Combination treatment was more effective than Nifedipine alone. *Karya et al. 2021* [90]
Calcium channel blockers + Progesterone	Dydrogesterone + Nifedipine (Combination vs. Nifedipine)	Total = 48 (24 vs. 24)	(< 34 weeks) 16.7% vs. 12.5% (*p* > 0.05) (< 37 weeks) 33.3% vs. 37.5% (*p* > 0.05)	32.7 days vs. 38.2 days (*p* > 0.05)	Adjuvant tocolysis of Dydrogesterone with acute tocolysis of Nifedipine did not improve the PTB rate. *Areeruk et al. 2016* [92]
	Nifedipine + progesterone (Combination vs. Nifedipine)	Total = 60 (30 vs. 30)	No information	(Prolongation of gestation until 36 weeks) 36.66% vs. 76.6% (*p* = 0.0001)	Combination treatment was more effective than Nifedipine alone. *Nisa et al. 2016* [93]
